# A Case of Post-Colonoscopy Cecal Perforation in a 78-Year-Old Man Responding to Conservative Management

**DOI:** 10.7759/cureus.22364

**Published:** 2022-02-18

**Authors:** Mohammed H Basendowah, Sahar A Futayni, Raghad A Ismail, Hussam A Alhazmi, Abdullah M Almatrafi, Ammar Y Hassan, Mohammed A Ashour

**Affiliations:** 1 Surgery, King Abdulaziz University Hospital, Jeddah, SAU; 2 Surgery, Alnoor Specilaist Hospital, Makkah, SAU; 3 College of Medicine, King Abdulaziz University, Jeddah, SAU

**Keywords:** iatrogenic colon perforation, iatrogenic cecal perforation, colon perforation conservative management, cecal perforation conservative management, conservative management, post-colonoscopy colon perforation, post-colonoscopy cecal perforation, post-colonoscopy complications, cecal perforation, colon perforation

## Abstract

Since the 1960s, colonoscopy has been the most extensively utilized diagnostic technique for colorectal cancer, and it is also a treatment tool for several colorectal diseases. Like many other medical treatments, it has complications, some of which might pose a major threat to the patient's health and wellbeing if not detected early enough. There is no consensus on the best way to treat colonoscopic perforation, and the majority of cases need immediate surgery. However, iatrogenic colon perforation can sometimes be treated conservatively. In this report, we describe a case of post-colonoscopic cecal perforation that was managed conservatively.

## Introduction

A colonoscope is a flexible endoscope that is used to visualize the colon and the terminal ileum. It was first introduced in clinical practice in the 1960s and has since become the primary approach for colorectal disease screening, diagnosis, and management [1,2]. Colonoscopies are relatively safe, although there is a risk of gastrointestinal bleeding, intraabdominal organ injury, cardiopulmonary disorders, and colon perforation (CP) in only eight of 10,000 cases [1,3]. Although it is rare, CP is considered one of the most serious complications and has significant morbidity and mortality rates, which reach as high as 53% and 25%, respectively [4]. The incidence of iatrogenic CP ranges from 0.03 to 0.8% for diagnostic colonoscopy and from 0.15 to 3% for therapeutic colonoscopy. The major risk factors for post-colonoscopy CP include female gender, age, multiple comorbidities, inflammatory bowel disease, and previous colon surgery [1]. 

Regarding the peritoneum, there are three different types of CP: intraperitoneal, extraperitoneal, and combinations. The majority of CPs are intraperitoneal, which allow air and intracolonic contents to flow into the peritoneal cavity. CP can occasionally be extraperitoneal, which allows air to enter the retroperitoneal space [5]. Free air flows in various anatomical regions depending on whether the perforation is intraperitoneal, extraperitoneal, or combined, which each generate clinical symptoms and signs [1].

Both medical and surgical approaches are utilized to manage CP. Surgical repair by either laparotomy or laparoscopy is the conventional treatment for iatrogenic perforation. Most cases require urgent surgery, but some can be managed conservatively. The choice depends on several factors, such as the patient's age, general health, comorbidities, and most critically, the period between the onset and diagnosis of perforation [6,7]. We present a case of post-colonoscopically cecal perforation that has been managed conservatively at King Abdulaziz University Hospital (KAUH), Jeddah.

## Case presentation

The patient is a 78-year-old male with a medical history of diabetes mellitus, hypertension, end-stage renal disease, intestinal arteriovenous malformation (AVM), and hidradenitis suppurativa. He presented to the emergency department (ED) with a complaint of sudden shortness of breath for one day. The dyspnea was exacerbated by movement and worsened over time. The patient could not speak in full sentences, and oxygenation at home failed due to the severity of his condition. The dyspnea was associated with dizziness, headache, and easy fatigability. The patient had a history of laparoscopic cholecystectomy 20 years prior. The physical examination was unremarkable except for the presence of significant pallor on the conjunctiva.

Laboratory investigations were performed, which showed significantly low levels of hemoglobin (8.2 g/dL) and RBCs (3.10 million cells/µL) according to a complete blood count test. An upper single balloon enteroscopy and colonoscopy were performed for the evaluation of anemia and the possible presence of bleeding. The balloon enteroscopy showed duodenal and jejunal AVM with argon beam coagulation. In addition, the colonoscopy showed right-sided diverticular disease and multiple small-sized telangiectasias in the cecum, which were managed endoscopically in the same manner.

By one day after the colonoscopy, the patient developed sudden, sharp, non-radiating abdominal pain in the lower-right quadrant. The patient was hemodynamically stable, and abdominal examination showed no signs of peritonitis, so an abdominal CT scan with oral contrast was performed. The results showed a thickening around the terminal ileum and base of the cecum secondary to sealed cecal perforation with fat stranding and an air pocket. The deepest pocket thickness was about 2 cm, and it contained fluid and gas, which suggested leakage.

The plan was to manage the patient conservatively by keeping him on nothing by mouth (NPO) and total parenteral nutrition (TPN) while maintaining the administration of intravenous tazobactam and vancomycin for seven days. During the management period, the patient was stable with no complications except for the development of one spike of fever. After seven days, an abdominal CT scan with oral contrast was repeated, which showed a mild interval decrease in the peri-cecal collection with fewer gas bubbles and no contrast. The deepest pocket thickness had become about 1.7 cm. Moreover, there were no other significant interval changes. On the same day, tazobactam was switched to metronidazole and ciprofloxacin.

**Figure 1:**
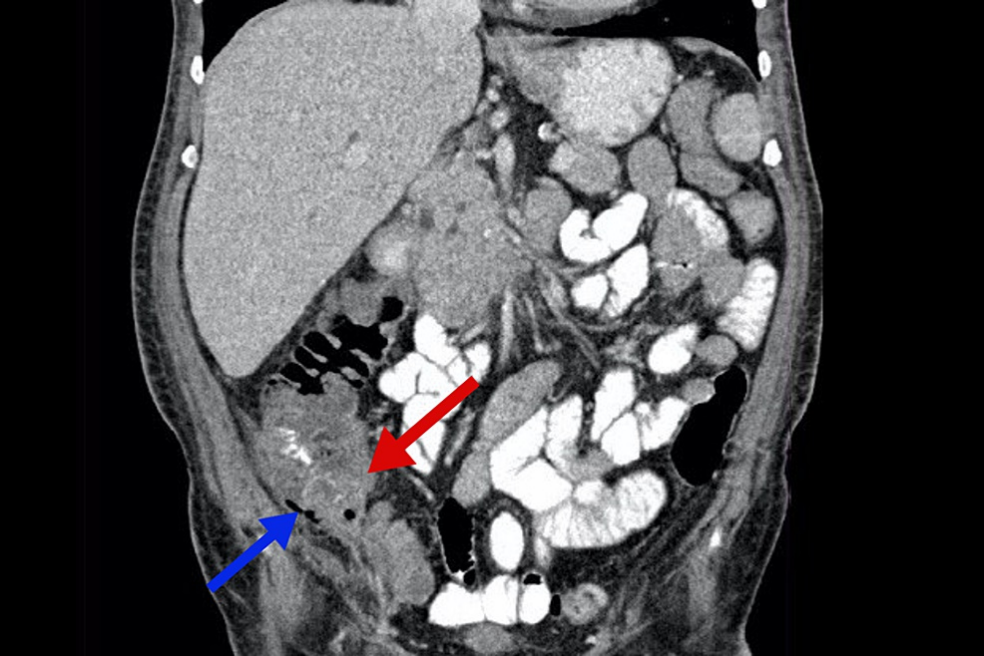
Coronal oral contrast CT scan of the abdomen and pelvis showing pericecal collection of fluids and gases, which is suggestive of leakage The red arrow points to the pericecal fluid collection, while the blue indicated the gas collection.

At present, the patient has been discharged in stable condition with no abdominal pain, nausea, or vomiting, and he is tolerating orally. Follow-up for clinical and imaging evaluation was scheduled after one week, and a repeated abdominal CT scan was performed. The scan showed a significant decrease in the pericecal collection, and the deepest pocket thickness was 1 cm (Figure 1).

## Discussion

Colonoscopy is a safe procedure that is considered the gold standard modality for colon cancer screening, along with other therapeutic and diagnostic indications. Like all types of procedures, it carries a risk of complications-most commonly CP, in addition to bleeding and cardiovascular morbidities. The risk of perforation is slightly higher in colonoscopies performed for therapeutic purposes (3%) than in diagnostic colonoscopies (0.8%) [1,2]. Other risk factors observed for increased risk of colonic perforation during colonoscopy are older age (>75 years), female gender, multiple comorbidities, the performance of colonoscopic interventions (e.g., biopsy, polypectomy, endoscopic mucosal resection, and endoscopic submucosal dissection), the presence of underlying intestinal pathology (e.g., inflammatory bowel disease), and malnutrition (low body mass index, low plasma albumin level) [1,2,8].

**Figure 2:**
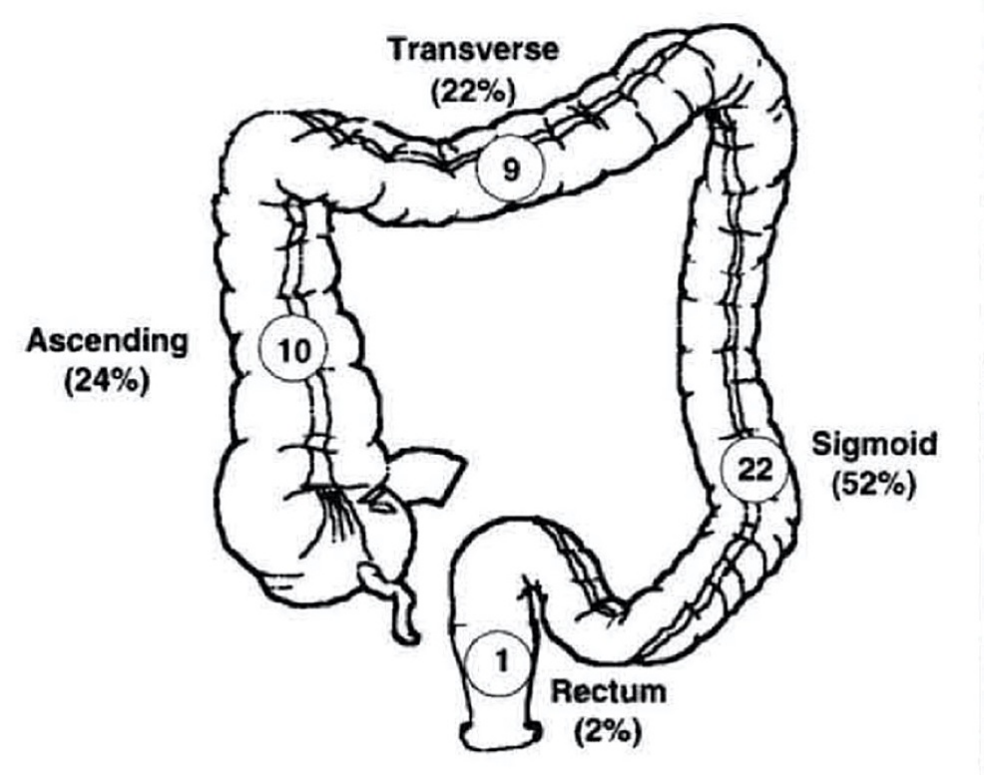
Site of colonic perforation Circled numbers = number of patients in the original study (n=42) [10] Reprinted with permission from David R. Farley et al. [10], license number: 5237841348691

Intraperitoneal CP may manifest with abdominal pain, peritonitis, and signs of acute abdomen. Extraperitoneal perforation may manifest with subcutaneous emphysema where free air spreads to the abdominal space, subcutaneous space, mediastinum, etc. Due to the continuity in the fascial planes, post-colonoscopy follow-up is important [1,9]. The presence of combined extra- and intraperitoneal perforation is also possible. The presence of abdominal pain and distention in a patient recently post-colonoscopy is considered CP until proven otherwise [2]. The most frequent area of CP is the sigmoid (52%), followed by the ascending (24%) including the cecum, with much less frequency in the transverse colon and rectum (Figure 2). 

**Figure 3:**
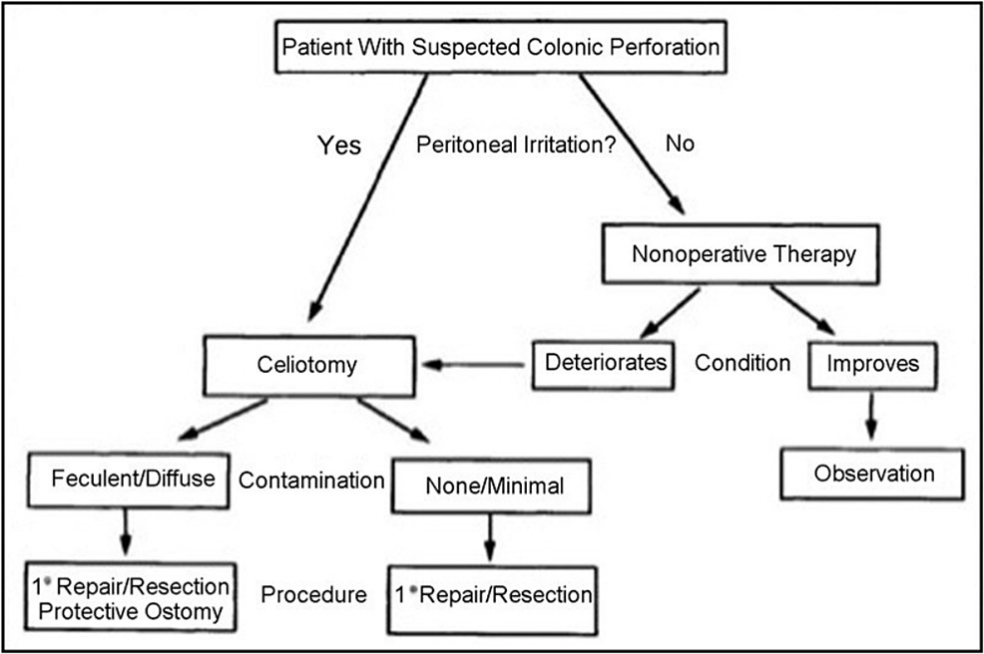
Management algorithm for colonoscopic perforation Reprinted with permission from David R. Farley et al. [10], license number: 5237841348691

In the management of CP in an unstable patient, prompt surgical intervention is warranted as shown in (Figure 3). The options include primary repair, resection with primary anastomosis, creation of a stoma, or in some cases, primary anastomosis with diverting stoma (ileostomy vs. colostomy). The choice depends on the condition of the patient and bowel, as well as the degree of contamination [2]. In stable patients with an absence of signs of generalized peritonitis, conservative management is possible in the form of bowel rest and broad-spectrum antibiotics with or without endoluminal repair [2]. 

## Conclusions

Colonoscopy is a widely used and safe procedure. The risk of complications is low, but they can be life-threatening. Thus, colonoscopy in elderly patients with multiple comorbidities should be performed with caution, given that the highest number of complications is reported in this age group. The conservative management of CP in a stable patient post-colonoscopy is an acceptable option, but it must be accompanied by close observation for any signs of deterioration.
